# Spectrum-Effect Relationships between High-Performance Liquid Chromatography (HPLC) Fingerprints and the Antioxidant and Anti-Inflammatory Activities of Collagen Peptides

**DOI:** 10.3390/molecules23123257

**Published:** 2018-12-10

**Authors:** Junwen Wang, Dan Luo, Ming Liang, Ting Zhang, Xiquan Yin, Ying Zhang, Xiangliang Yang, Wei Liu

**Affiliations:** 1 College of Life Science and Technology, Huazhong University of Science and Technology, Wuhan 430074, China; wangjw@hust.edu.cn (J.W.); yangxl@hust.edu.cn (X.Y.); 2 National Engineering Research Center for Nanomedicine, Wuhan 430075, China; Laurel565118@163.com; 3 Infinitus (China) Co., Ltd., Guangzhou 510665, China; Fiona.Liang@infinitus-int.com (M.L.); Tinca.Zhang@infinitus-int.com (T.Z.); Xiquan.Yin@infinitus-int.com (X.Y.); sophia.zhang@infinitus-int.com (Y.Z.)

**Keywords:** collagen peptide, HPLC fingerprint, antioxidant, anti-inflammatory, spectrum-effect relationship

## Abstract

A total of 13 batches of collagen peptide samples were extracted, isolated, and purified from chicken sternal cartilage under various process parameters. The fingerprint profiles of 13 batches of collagen peptides were established by high-performance liquid chromatography (HPLC). In addition, the amino acid profiles and molecular weight distributions of collagen peptides were investigated. The in vitro antioxidant activities of the peptide samples were measured using the 2,2′-Azinobis (3-ethylbenzothiazoline-6-sulphonic acid) diammonium salt (ABTS) assay, the 2,2-diphenyl-1-picrylhydrazyl (DPPH) assay, the ferric-reducing antioxidant power (FRAP) assay and an assay of the oxidative damage induced by hydrogen peroxide (H_2_O_2_) in the degenerative cartilage cells from the knee joint of rat C518 (C518 cell line). The anti-inflammatory activities of the peptide samples were assessed by measuring the inflammatory responses induced by lipopolysaccharides (LPS) in C518 cells. Subsequently, the spectrum-effect relationships between HPLC fingerprints and the antioxidant and anti-inflammatory activities of collagen peptides were investigated using grey relational analysis (GRA). Fifteen common peaks were obtained from the HPLC fingerprints of collagen peptides. Each collagen peptide sample had a characteristic set of amino acid types and contents. All of the hydrolysates of the collagen peptides were primarily composed of fractions II (500–1000 Da) and III (1000–3000 Da). Collagen peptides exhibited good scavenging activity on ABTS radical, DPPH radical, and ferric-reducing antioxidant power. Collagen peptides were also effective against H_2_O_2_-induced cellular oxidative damage in C518 cells. The antioxidant activity of collagen peptides was due to the low molecular weight and the presence of antioxidant and hydrophobic amino acid residues within its sequence. Collagen peptides significantly inhibited the secretion of inflammatory cytokines IL-1β, TNF-α, and PGE2 in C518 cells. The anti-inflammatory activity of collagen peptides may include increased synthesis of the key components of extracellular matrix (ECM) and inhibited apoptosis of chondrocytes. The GRA results showed that peaks 2, 3, and 8 were the main components contributing to the antioxidant activity of the collagen peptides, whereas peaks 11 and 14 were the main components contributing to the anti-inflammatory activity of the collagen peptides. The components of peaks 8 and 14 were identified as GPRGPPGPVGP and VAIQAVLSLYASGR by UPLC-MS/MS. Those identified collagen peptides offer a potential therapeutic strategy for the treatment of osteoarthritis (OA) due to their antioxidative stress and due to them disturbing the catabolism and anabolism processes in arthrodial cartilage.

## 1. Introduction

Osteoarthritis (OA), the most common degenerative joint disease worldwide found in elderly individuals, is a leading cause of physical disability. Type II collagen (CII) is a principal component of the extracellular matrix (ECM) and constitutes 90–95% of the total protein content in the articular cartilage [1]. Repeated oral administrations of CII induce oral tolerance and inhibit the development of OA. The immunologic response to orally administered antigens occurs in the gut-associated lymphoid tissue (GALT), which is composed of epithelium, lamina propria, Peyer’s patch (PP), and mesenteric lymph nodes (MLN). Orally taken native CII antigens interact with PP in the GALT, resulting in turning off the T-cell attack to CII in the cartilage. This desensitization process in PP, also known as oral tolerance, avoids the recognition of endogenous CII in the cartilage as an antigen by the immune system [2]. The effects of oral administration of CII obtained from chicken, bovine, and sheep sources have been evaluated for the treatment of arthritis [3]. Chicken sternal cartilage containing a high amount of collagen is recognized as a potential source of CII [4]. Naturally derived CII from chick sternal cartilage is thought to be a potential oral alternative for OA treatment.

Although the etiology and underlying mechanisms of OA are complicated, much evidence has suggested that the progression of OA in patients is significantly associated with oxidative stress and the inflammatory factor network [5,6].

Recently, oxidative stress has been one of the research hotspots in the field of OA. There is increasing evidence that oxidative stress due to chronic production of endogenous reactive oxygen species (ROS) plays an important role in the physiology and pathophysiology of OA. Oxidative stress regulates intracellular signaling processes and induces chondrocyte senescence and apoptosis, which is characterized by degradation of ECM and a decrease in the synthesis of proteoglycan and CII [7]. Moreover, increased levels of ROS can damage DNA, including mitochondrial DNA, thereby affecting cell viability and contributing to the disruption of ECM homeostasis. Increased levels of ROS may also contribute to the senescent secretory phenotype and the reduced sensitivity of chondrocytes to insulin-like growth factor I (IGF-I) [8].

Antioxidant properties should be evaluated by a variety of methods because most natural antioxidants are multifunctional. Several assays have been frequently used to estimate antioxidant capacities in natural antioxidants, including the 2,2′-Azinobis (3-ethylbenzothiazoline-6-sulphonic acid) diammonium salt (ABTS) assay, the 2,2-diphenyl-1-picrylhydrazyl (DPPH) assay, and the ferric-reducing antioxidant power (FRAP) assay [9]. H_2_O_2_ is a common ROS produced in cellular metabolism. Intracellular steady-state concentrations of H_2_O_2_ above 1 μM cause oxidative stress that induces growth arrest and cell death. H_2_O_2_ induces chondrocyte apoptosis through the regulation of phosphatidylinositol 3-kinase (PI3K)/protein kinase B (Akt) and c-Jun N-terminal kinase (JNK) signaling pathways [5]. In experimental models used to investigate oxidative stress responses of cells, or cytoprotection by antioxidant agents, cultured cells are often exposed to H_2_O_2_ added as a bolus into the culture medium [10]. Therefore, the ABTS assay, DPPH assay, FRAP assay and an assay of cellular antioxidant activity (the protective effect of collagen peptides against H_2_O_2_-induced cellular oxidative damage in C518 cells) were performed to evaluate the antioxidant activity of collagen peptides.

Inflammation is another promoting factor in the OA process, including chondrocyte and synovium inflammation. The group of inflammatory cytokines is the most important group of compounds participating in the pathogenesis of OA. Among the many representatives of this group, the greatest importance is attributed to IL-1β, TNF-α, and PGE2, which have been shown to modulate ECM turnover, to accelerate the degradation of cartilage and to induce chondrocyte apoptosis in the development of OA [6,11].

IL-1β demonstrates potent bioactivities in inhibiting ECM synthesis and promoting cartilage breakdown [12]. The matrix metalloproteinases (MMPs) play important roles in cartilage degradation in OA, and IL-1β significantly upregulates the expression of MMPs, aggravating degradation processes in OA [13]. Moreover, it has been suggested that IL-1β induces the expression of the TNF-α gene in chondrocytes and upregulates the surface expression of the TNF receptor (TNFR) [14]. The effect of TNF-α in most cases coincides with the action of IL-1β, and in the case of many phenomena occurring in the course of OA there is a marked synergism between the two cytokines. This effect is the result of activation of the same group of intracellular signaling pathways, which in turn triggers similar effects that increase the inflammation and catabolism in joint tissues [6]. PGE2 is considered to be the major contributor to inflammatory pain in arthritic conditions. IL-1β has been shown to stimulate and produce high levels of PGE2 that may induce pain and degeneration with OA [12]. Pharmacologic inhibitors to these mediators may potentially be used as biological treatments in the future. Accordingly, the protective effect of collagen peptides against lipopolysaccharide (LPS)-induced proinflammatory mediators IL-1β, TNF-α, and PGE2 in C518 cells was investigated.

In virtue of the extreme complexity of collagen peptides, the components contributing to their effects are still unclear. Traditional methods for analysis of the active components of peptides are complex and time-consuming, and the spectrum-effect relationships of collagen peptides have not been reported. The aim of this study was to develop an efficient method for screening the active components of CII by establishing spectrum-effect relationships. 

## 2. Results and Discussion

### 2.1. High-Performance Liquid Chromatography (HPLC) Fingerprints of the Collagen Peptides

**Figure 1:**
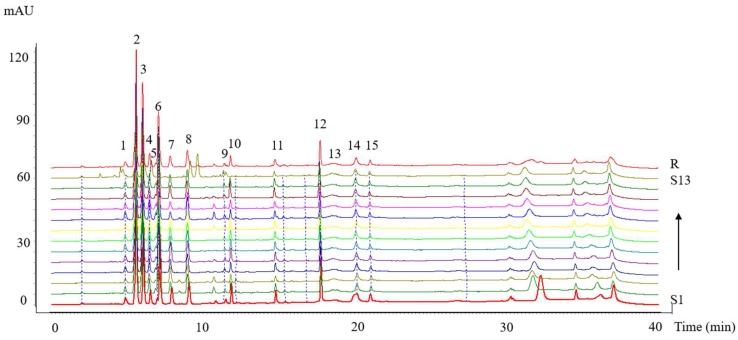
The high-performance liquid chromatography (HPLC) fingerprints of 13 batches of collagen peptide samples (S1–S13) and reference standard fingerprint (R).

The HPLC fingerprints of the collagen peptides and the reference chromatogram generated by the software are shown in Figure 1. The HPLC fingerprints of collagen peptide samples S1–S13 are shown in Figures S1–S13. Fifteen common peaks were obtained from all chromatograms, which were marked 1–15 in the reference chromatogram. Peak 8 (*t* = 9.087 min) was selected as the reference peak because it was a medium peak in the middle of the chromatogram. The relative retention time (RRT), relative peak area (RPA), and coefficient of variance (C.V.%) of the peak area of 15 common characteristic peaks are shown in Table 1. The majority of the C.V.% values were greater than 18.54%, which showed that the content of each common constituent in the samples varied significantly. The content of the unknown component, which was represented by peaks 5 and 7, showed a particularly large degree of variation.

The similarity values ranged from 0.612 to 0.998 (Table 2). Compared to the reference chromatogram, the similarities between the reference fingerprint and the entire chromatographic profiles of 13 batches of collagen peptide samples were evaluated. The correlation coefficients of batches 1–13 were 0.777, 0.936, 0.972, 0.963, 0.977, 0.976, 0.969, 0.977, 0.972, 0.967, 0.961, 0.953, and 0.711, respectively. The similarity values of all batches exceeded 0.930, with the exception of S1 and S13, which indicated that there were relatively small differences among them.

### 2.2. HPLC Fingerprints of Amino Acids

**Figure 2:**
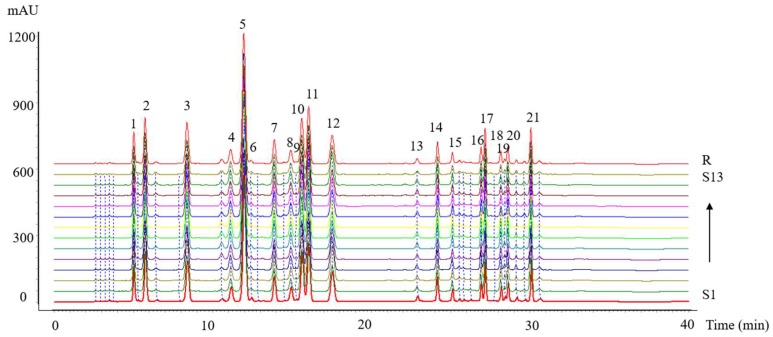
The HPLC fingerprints of the amino acids of 13 batches of collagen peptide samples (S1–S13) and amino acid reference standard fingerprint (R).

The HPLC fingerprints of the amino acids and the reference chromatogram generated by the software are shown in Figure 2. Twenty-one common peaks were obtained from all chromatograms, which were marked 1–21 in the reference chromatogram. Peak 7 (Arg, *t* = 13.881 min) was selected as the reference peak because it was a medium peak in the middle of the chromatogram. The RRT, RPA, and C.V.% of the peak area of 21 common characteristic peaks are shown in Table 3.

Subsequently, eighteen common characteristic peaks in the chromatogram were identified by comparing the retention times and online UV spectra with those of the standards as follows: Peak 1 (Asp), peak 2 (Glu), peak 4 (Ser), peak 5 (Gly), peak 6 (His), peak 7 (Arg), peak 8 (Thr), peak 9 (HyP), peak 10 (Ala), peak 11 (Pro), peak 13 (Tyr), peak 14 (Val), peak 15 (Met), peak 16 (Ile), peak 17 (Leu), peak 19 (HyL), peak 20 (Phe), and peak 21 (Lys). Each collagen peptide sample had a characteristic set of amino acid types and contents. As a whole, the collagen peptide samples were rich in Gly, Ala, and Pro, which accounted for 23.98%, 16.20%, and 9.64% of the amino acid residues, respectively, whereas HyP (0.24%), HyL (0.51%), His (0.52%), and Tyr (0.73%) were relatively rare among the 18 quantified amino acids (Table 3).

It was found that the collagen peptide samples had a similar amino acid composition to the reference. The characteristic that collagen peptide samples had a high content of Gly and Pro was in accordance with previous findings, although the contents of Gly and Pro obtained in the current research were slightly lower than the values reported by Cao [4]. From these results it can be concluded that Gly, which constitutes approximately one-fourth of all residues in collagen, was present as a quarter residue in the sequence, and that high amounts of Pro could be accommodated while maintaining planar peptide bonds. These assumptions led to the construction of the correct model of sternal cartilage collagen as a (Gly-X-Y) n pattern. Indeed, it was the most commonly found triplet in collagen chains space, indicating that the collagen purified from chicken sternal cartilage was typical CII. Bioactive peptides usually contain 3–20 amino acid residues, and their activities are related to their amino acid compositions [15]. Generally, the antioxidant activities of the amino acid side chain, such as the imidazole group in His (peak 6), the phenolic hydroxyl group in Tyr (peak 13), and thioether in Met (peak 15), determine the antioxidant activities of peptides [16]. Additionally, antioxidative peptides containing aromatic amino acids at the *C*-terminus have high radical scavenging activities. Aromatic amino acids such as His (peak 6) and Tyr (peak 13) increase the antioxidant activities of peptides and protein hydrolysates because they easily donate protons to electron-deficient radicals and maintain their stabilities via resonance structures. The hydrophobic amino acids at the N-terminus of a peptide sequence, such as Gly (peak 5), Val (peak 14), and Leu (peak 17), are believed to contribute to antioxidative activity, as it is thought that hydrophobic amino acids can increase the abundance of peptides at the water–lipid interface, which may facilitate greater interaction between the peptide and fatty acids [17,18]. Collagen peptide samples S3 and S13 exhibited a lower abundance of Gly, His, Leu, Met, Tyr, and Val in comparison to the other samples. These findings were in accordance with the results of the ABTS and DPPH assays, which indicated that low antioxidant amino acid content in the collagen peptide samples might be related to low antioxidant activity.

The similarity values ranged from 0.995 to 1.000 (Table 4). Compared to the reference chromatogram, the similarities between the reference fingerprint and the entire chromatographic profiles of 13 batches of collagen peptide samples were evaluated: The correlation coefficients of batches 1–13 were 0.999, 1.000, 0.999, 0.999, 0.998, 0.999, 0.999, 0.999, 0.999, 1.000, 0.999, 0.999, and 0.999, respectively. The similarity values of all batches exceeded 0.998, which indicated that there were relatively small differences among them.

### 2.3. Molecular Weight Distribution of Cartilage Hydrolysates

The collagen peptides were fractionated according to molecular weight into four categories: I (below 500 Da), II (500–1000 Da), III (1000–3000 Da), and IV (heavier than 3000 Da). The relative content, obtained from their peak areas relative to the total peak area, of each peptide fraction is presented in Table 5. It was found that all of the hydrolysates were primarily composed of fractions II (500–1000 Da) and III (1000–3000 Da), which accounted for approximately 64.80% of the sample after enzymatic treatment. 

Molecular weight reflects the hydrolysis of proteins and is highly correlated with the bioavailability and bioactivity of peptides [19,20]. Peptides of low molecular weight are more potent than those of high molecular weight because they cross the intestinal barrier more easily and exert biological effects. Moreover, short-chain peptides are more effective than long-chain peptides with regard to their effects on physiological processes, as they are less susceptible to gastrointestinal hydrolysis. 

The peptide fraction with lower molecular weight was probably associated with higher antioxidant activity. Samples S1, S10, and S11 had greater percentages of fractions I (below 500 Da) and II (500–1000 Da) in comparison to the other samples. In contrast, S13 contained relatively low percentages of fractions I (below 500 Da) and II (500–1000 Da) (Table 5). These findings were consistent with observations from the in vitro antioxidant assay mentioned below and support the idea that the functional properties of antioxidative peptides are highly influenced by their molecular weight. 

### 2.4. Antioxidant Activity

#### 2.4.1. Comparative Antioxidant Activity of 13 Batches of Collagen Peptides

The ABTS radical scavenging activity of the collagen peptides varied from 34.91% to 48.14%, which represented a variation of approximately 1.4-fold. The results showed that the ABTS radical inhibitory capacity of the samples (at a concentration of 2 mg/mL) decreased in the following order: S1 > S11 > S7 > S8 > S5 > S12 > S2 > S4 > S10 > S6 > S9 > S3 > S13. S1 exhibited the most ABTS radical scavenging activity, followed by S11 and S7 (Table 6).

The DPPH radical scavenging activity of the collagen peptides varied from 18.46% to 55.06%, which represented a variation of approximately three-fold. The results showed that the DPPH radical inhibitory capacity of the samples (at a concentration of 25 mg/mL) decreased in the following order: S12 > S11 > S10 > S5 > S7 > S9 > S6 > S8 > S4 > S2 > S1 > S3 > S13. S12 exhibited the most DPPH radical scavenging activity, followed by S11 and S10 (Table 6).

The ferric radical scavenging activity of collagen peptides varied from 31.56% to 49.10%, which represented a variation of approximately 1.5-fold. The results showed that the ferric radical scavenging activity of the samples (at a concentration of 50 mg/mL) decreased in the following order: S10 > S9 > S3 > S11 > S6 > S1 > S2 > S7 > S12 > S8 > S4 > S5 > S13. S10 exhibited the most ferric radical scavenging activity, followed by S9 and S3 (Table 6).

The results of these experiments suggested that all of the collagen peptide samples possessed good in vitro antioxidant efficacy. Furthermore, previous results suggested that the chicken sternal cartilage hydrolysates had great oxygen radical absorption capacity (ORAC), with a half maximal inhibitory concentration (IC50) value of 0.48 ± 0.02 μM TE/mg peptides. The exposure of abundant antioxidant residues was responsible for their ORAC antioxidant activity [21]. It is interesting to note that S13 exhibited the lowest antioxidant capacity in all three antioxidant assays, although the antioxidant activity of the collagen peptides varied according to the assay used.

The enzyme/substrate (E/S) ratio, pH, and temperature of digestion had a more significant effect on the hydrolysis of collagen peptides compared to digestion time, as no significant difference of the DPPH radical scavenging activity of S12, S11, and S10 was observed with the extension of the hydrolysis time.

It was clear that enzyme dosage was an important parameter for collagen peptide hydrolysis, since there were relatively significant differences in the antioxidant activity of S10 and S13, which was particularly evident in the FRAP assay.

S11 exhibited the greatest antioxidant capacity in the ABTS assay, DPPH assay, and FRAP assay (the top 4). Thus, the optimal enzymatic hydrolysis conditions for collagen peptide, papain treatment at an E/S ratio of 0.50% at 50 °C and at pH 6.5 for 5 h, could be used as a basis for production scale-up. The results of the optimal enzymatic hydrolysis conditions were basically consistent with a previous study [22].

#### 2.4.2. Protective Effect of Collagen Peptides against H_2_O_2_-Induced Oxidative Damage

**Figure 3:**
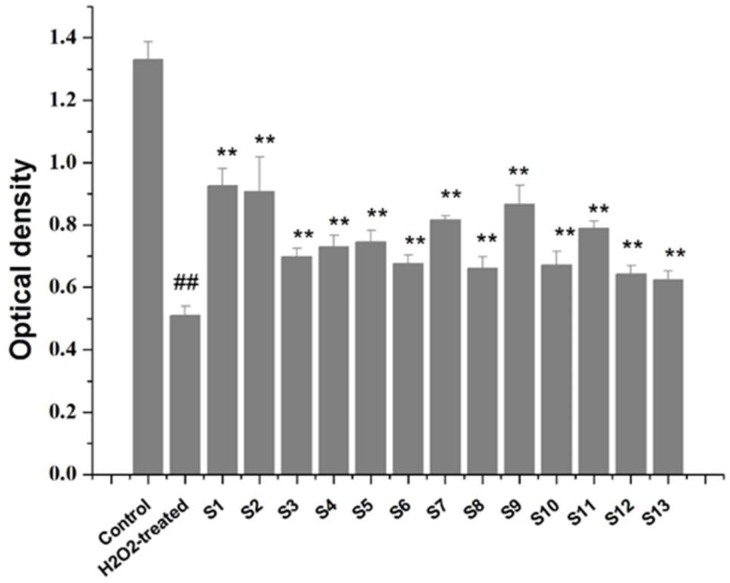
Collagen peptides prevented H_2_O_2_-induced oxidative damage. Data are expressed as means ± SD (*n* = 3); ** *p* < 0.01 versus H_2_O_2_-treated group; ^##^ *p* < 0.01 versus the control group.

The results of the Cell Counting Kit-8 (CCK-8) assay showed that the collagen peptides were not cytotoxic (*P* < 0.01). Exposure to exogenous H_2_O_2_ decreased the viability of C518 cells, which indicated that exposure to H_2_O_2_ was cytotoxic for C518 cells. In contrast, a significant increase in C518 cell viability was observed in all samples, especially for S1, S2, and S9, when C518 cells were pretreated with collagen peptides (Figure 3). Taken together, these findings clearly show that collagen peptides conferred significant protection against H_2_O_2_-induced cellular oxidative damage and can thus be used as a natural antioxidant.

### 2.5. Inhibitory Effects of Collagen Peptides on LPS-Induced Release of Proinflammatory Mediators IL-1β, TNF-α, and PGE2

The effects of the collagen peptide samples on IL-1β secretion varied significantly. S2, S10, and S11 exhibited extremely strong inhibitory effects on IL-1β expression, whereas those of S1 and S12 were slightly weaker. S8, S9, and S13 showed strong inhibitory effects on IL-1β expression, whereas S3, S4, S5, S6, and S7 did not show anti-inflammatory activity (Table 7).

The levels of released TNF-α were increased in the LPS-stimulated culture media. Pretreatment with collagen peptides at a concentration of 2.0 mg/mL for 24 h substantially blocked TNF-α generation in C518 cells. S1, S2, and S3 showed the strongest inhibitory effects on TNF-α generation (Table 7).

All collagen peptide samples significantly attenuated LPS-induced PGE2 production, which was not consistent with the data from the experiments assessing IL-1β and TNF-α secretion. S1, S2, and S8 showed the strongest inhibitory effects on LPS-induced PGE2 production (Table 7).

Taken together, our findings show that collagen peptides significantly inhibited the secretion of IL-1β, TNF-α, and PGE2. Notably, the inhibitory effect of collagen peptides on PGE2 was particularly apparent. Furthermore, previous studies also confirmed that CII treatment dose-dependently inhibited the overproduction of inflammatory cytokines levels (IL-1β and TNF-α) in synoviocytes in the collagen-induced rat arthritis (CIA) model. In conclusion, CII extracted from chick sternal cartilage possessed antiarthritis activity, which may have been a result of its regulation of the humoral and cellular immune systems [23]. Thus, downregulation of proinflammatory mediators by collagen peptides may be a promising therapy for OA. Nevertheless, the molecular mechanisms underlying the anti-inflammatory effects of collagen peptides are not completely understood, so further investigation is required.

### 2.6. GRA Results

The ranking results of the GRA were as follows (the top 10): ABTS scavenging activity, 14 > 8 > 15 > 3 > 6 > 2 > 1 > 13 > 5 > 4; DPPH scavenging activity, 2 > 8 > 6 > 13 > 3 > 12 > 10 > 14 > 5 > 11; ferric reducing antioxidant power, 2 > 3 > 8 > 1 > 4 > 14 > 6 > 15 > 13 > 11; protection against H_2_O_2_-induced oxidative damage, 10 > 11 > 15 > 14 > 9 > 2 > 12 > 1 > 4 > 5; inhibited production of LPS-induced IL-1β, 11 > 12 > 10 > 14 > 15 > 9 > 3 > 8 > 5 > 1; inhibited production of LPS-induced TNF-α, 3 > 14 > 8 > 11 > 15 > 2 > 7 > 12 > 10 > 9; inhibited production of LPS-induced PGE2, 6 > 14 > 8 > 2 > 3 > 15 > 5 > 11 > 1 > 12 (Table 8). Overall, peaks 2, 3, and 8 were the main contributors to the antioxidant activity of collagen peptides, whereas peaks 11 and 14 were the main contributors to the anti-inflammatory activity of collagen peptides. 

Generating peptides via proteolysis or peptidolysis results in complex combinations of peptides with different masses and physicochemical properties. Classical strategies commonly used to achieve isolation and subsequent characterization employ chromatographic techniques coupled to mass spectrometry, in particular liquid chromatography (LC) coupled to tandem MS (MS/MS) [24,25]. The accuracy and speed of peptide identification are some of the key features that set MS/MS apart from the other methodologies used to analyze protein mixtures [26]. Moreover, the probability-based search engine Mascot has found widespread use as a tool in correlating tandem mass spectra with peptides in a sequence database [27,28].

The amino acid sequences of the collagen peptides were tentatively identified using ultra-performance liquid chromatography-tandem mass spectrometry (UPLC-MS/MS), and all the resulting fragmentation spectra (MS/MS) were searched against the *Gallus gallus* database (NCBI) with the Mascot database searching software (version 2.1, Matrix Science, London, UK). In addition, we also compared the ultraviolet spectra and retention times of the peaks with those of standard peptides obtained by the solid phase peptide synthesis (SPPS) method, and they matched each other. The primary structures of the peptide fractions were determined as GPRGPPGPVGP (peak 8, 987.12 Da) and VAIQAVLSLYASGR (peak 14, 1447.67 Da). Peaks 8 and 14 probably each represented more than one peptide, but only the structure of the main component of each peak was identified in this study. This is the first report of the isolation and identification of these peptides from chicken sternal cartilage. However, further study is needed to identify the structures of other collagen peptide components, particularly the effective components.

## 3. Materials and Methods

### 3.1. Materials

HPLC-grade acetonitrile was purchased from Tedia (Fairfield, OH, USA). Amino acid standards, ABTS, DPPH, 2,4,6-tripyridyl-s-triazine (TPTZ), LPS, CCK-8, rat IL-1β, PGE2, and the TNF-α ELISA kit were purchased from Sigma-Aldrich (St. Louis, MO, USA). Dulbecco’s Modified Eagle’s Medium (DMEM), fetal bovine serum (FBS), phosphate-buffered saline (PBS), and 1% penicillin/streptomycin were bought from Gibco (Carlsbad, CA, USA). The synthesized peptides (purity ≥ 95%) were obtained from Bankpeptide Biological Technology Co., Ltd. (Hefei, China). Other reagents were of analytical grade.

### 3.2. Preparation of Collagen Peptides

Collagen peptide was prepared according to the methods reported by Xie with some modifications [22]. Fresh chicken sternal cartilage was removed from the periosteum and the calcified portion, cut into slices, minced into paste, and stored at −20 °C. Then, the paste was treated with 5 M guanidine hydrochloride in Tris-HCl (0.05 M, pH 7.5) at a sample/solution ratio of 1:10 (*w*/*v*), with gentle stirring for 24 h at 4 °C to remove proteoglycans [29]. The precipitate was washed using Tris-HCl (0.05 M, pH 7.5) and acetic acid (0.5 M). In the enzymatic hydrolysis process of collagen peptides, the four parameters including the enzyme (papain) to substrate ratio (E/S: 0.25%, 0.50%, and 0.75% *w*/*w*), pH (6.0, 6.5, and 7.0), temperature (50, 55, and 60 °C) and time (4, 5, and 6 h), were investigated in single-factor experiments (Table 9). At the end of the enzymatic hydrolysis, the solution was heated in a boiling water bath for 10 min to inactivate the enzymes. The extracts were collected by centrifugation (Avanti J-26XP, Beckman Coulter, Brea, CA, USA) at 10,000× *g* for 30 min at 4 °C. The supernatant was collected and salted out overnight at 4 °C by adding 2.5 M NaCl in Tris-HCl (0.05 M, pH 7.5) at a ratio of 1:20 (*w*/*v*). The resulting precipitate was collected by centrifugation (Avanti J-26XP, Beckman Coulter, USA) at 10,000× *g* for 20 min at 4 °C. Subsequently, the precipitate was dissolved with acetic acid (0.1 M) and allowed to reach dialysis equilibrium with NaCl (0.2 M). The dialysate was freeze-dried and referred to as “collagen peptides”.

### 3.3. HPLC Fingerprints

#### 3.3.1. HPLC Conditions

Chromatographic analyses of collagen peptides were performed using a Shimadzu LC-20A HPLC system (Kyoto, Japan), including a quaternary solvent delivery system, online degasser, autosampler manager, column compartment and diode-array detector (DAD), as well as LCsolution software. The HPLC operating conditions were as follows: The column was an Inertsil ODS-SP C18 column (4.6 × 250 mm, 5 μm). A binary gradient elution system, comprising buffer (A) 0.05% TFA/acetonitrile and buffer (B) 0.1% TFA/water, was applied as follows: Initial, 98% B; 30 min, 40% B; 30.01 min, 98% B; 40 min, 98% B. The detection wavelength was set at 214 nm, the flow rate was 0.8 mL/min, the column temperature was maintained at 40 °C, the sample temperature was ambient, and the injection volume was 10 μL.

#### 3.3.2. Preparation of the Sample Solution

Collagen peptides were dissolved in deionized water at a concentration of 10 mg/mL and then filtered through a 0.22 μm micropore film to yield the sample solution.

#### 3.3.3. Analysis of HPLC Fingerprints

##### Validation of Methodology

The chromatographic fingerprinting methodology was validated to assess its precision, repeatability, and stability. Precision was evaluated by the analysis of six injections of the same testing sample consecutively. Repeatability was examined by determination of six different samples prepared from the same collagen peptide sample. Stability was examined by analysis of the sample solution at 0, 2, 4, 6, 8, 12, and 24 h. The methodology validation showed that the relative standard deviation (RSD) for precision was in the range of 1.28–1.75%, whereas that of reproducibility was less than 1.18% and that of storage stability was 1.46–1.83%. All results indicated that the HPLC fingerprint analysis method was valid and satisfactory.

##### Similarity Analysis

The HPLC fingerprints were matched automatically using the Similarity Evaluation System for Chromatographic Fingerprint of Traditional Chinese Medicine software developed by the Chinese Pharmacopoeia Committee (Version 2012) (Beijing, China). The reference fingerprint was generated using the median method [30], after which the similarity values between the reference fingerprint and the entire chromatographic profiles of 13 batches of collagen peptides were calculated.

### 3.4. Amino Acid Composition

Amino acid composition of collagen peptides was determined according to the method of Sun with a slight modification [31]. A high-performance liquid chromatography equipped with a PICO.TAG column (Waters, Milford, MA, USA) was used. The amino acid composition of collagen peptides was determined after hydrolysis at 150 °C for 1 h with 6 M hydrochloric acid prior to derivatization with phenyl isothiocyanate. A binary gradient elution system, comprising (A) 0.1 M ammonium acetate (the pH was adjusted to 6.5 with acetic acid)/acetonitrile (93:7 ratio) and (B) 80% acetonitrile/water, was applied as follows: Initial, 0% B; 15 min, 15% B; 18 min, 24% B; 25 min, 40% B; 30 min, 40% B; 30.01 min, 100% B; 40 min, 100% B. The amino acid standards included l-alanine (Ala), l-arginine (Arg), l-aspartic acid (Asp), l-glutamic acid (Glu), l-glycine (Gly), l-histidine (His), hydroxylysine (HyL), hydroxyproline (HyP), l-isoleucine (Ile), l-leucine (Leu), l-lysine (Lys), l-methionine (Met), l-phenylalanine (Phe), l-proline (Pro), l-serine (Ser), l-threonine (Thr), l-tyrosine (Tyr), and l-valine (Val).

### 3.5. Molecular Weight Distribution

Molecular weight distribution of collagen peptides was determined by gel permeation chromatography on a Superdex Peptide HR 10/300 GL (10 × 300 mm, Amersham Biosciences Co., Piscataway, NJ, USA) with UV detection at 214 and 280 nm. The mobile phase was 5 mM phosphate buffer containing 10 mM NaCl (pH 7.4), at a flow rate of 0.5 mL/min, which corresponded to an operating pressure of 1.8 MPa. A molecular weight calibration curve was obtained from the following standards: Glycine trimer (189 Da), oxidized glutathione (612 Da), vitamin B12 (1355 Da), aprotinin (6500 Da), cytochrome C (12500 Da), and bovine serum albumin (66430 Da) (Sigma Co., St. Louis, MO, USA). UNICORN 5.0 software (Amersham Biosciences Co., Piscataway, NJ, USA) was used to analyze the chromatographic data.

### 3.6. Antioxidant Activity Determination

#### 3.6.1. ABTS Assay

The ABTS assay was conducted following the method of Arnao with some modifications [32]. The stock solutions included 7.0 mM ABTS^+^ solution and 2.45 mM K_2_S_8_O_2_ solution. The working solution was prepared by mixing the two stock solutions in equal quantities and allowing them to react for 12 h at room temperature in the dark. Before analysis, this stock solution was diluted with PBS to obtain an absorbance of 0.70 ± 0.02 at 734 nm. Next, 100 μL samples were allowed to react with 2 mL ABTS·^+^ solution in the dark. After 6 min, the absorbance was recorded at 734 nm. Results were determined using the following Equation (1):

ABTS radical scavenging capacity (%) = [(A_blank_ − A_sample_)/A_blank_] × 100%, (1)

where A_sample_ and A_blank_ are the absorbance of the test sample and blank sample, respectively.

#### 3.6.2. DPPH Assay

The DPPH assay was performed according to the method of Brand–Williams with some modifications [33]. First, 1 mL of 0.2 mM DPPH solution was added to an equal volume of the sample solution. The mixture was vortexed for a few seconds and incubated for 30 min at room temperature, after which the absorbance of the mixture was measured at 517 nm. Results were calculated according to the following Equation (2):

DPPH radical scavenging activity (%) = [(A_blank_ − A_sample_)/A_blank_] × 100%, (2)

where A_sample_ and A_blank_ are the absorbance of the test sample and blank sample, respectively.

#### 3.6.3. FRAP Assay

The FRAP assay was performed according to the methods reported by Benzie with some modifications [34]. The FRAP solution was prepared by mixing 10 volumes of acetate buffer (300 mM, pH 3.6) with 1 volume of TPTZ (10 mM dissolved in 40 mM HCl) and 1 volume of FeCl_3_·6H_2_O (20 mM in water). Next, 200 μL of the sample were allowed to react with 2 mL FRAP solution in the dark for 30 min at 37 °C, and the absorbance of the sample was recorded at 593 nm. Aqueous standard solutions of FeSO_4_·7H_2_O (100–1000 μM) were used to generate a calibration curve. The final results were expressed as millimole FeSO_4_·7H_2_O equivalents per gram of collagen peptides (mM FeSO_4_·7H_2_O/g, collagen peptides).

#### 3.6.4. In Vitro H_2_O_2_-Induced Oxidative Damage Model

##### Cell Lines and Culture

C518 cells were purchased from Saiqi Biological Engineering Co., Ltd. (Shanghai, China). C518 cells were cultured and maintained in DMEM supplemented with 10% FBS, 100 U/mL penicillin, and 100 μg/mL streptomycin at 37 °C under a humidified atmosphere containing 5% CO_2_.

##### Protective Effects of Collagen Peptides against H_2_O_2_-Induced Oxidative Stress

C518 cells were seeded in 96-well plates (2 × 10^4^ cells/mL, 100 µL) and incubated for 24 h. They were pretreated with various collagen peptide samples (1.0 mg/mL) and cultured for 30 min. The control was pretreated with DMEM under the same conditions. Thereafter C518 cells were exposed to H_2_O_2_ (1 mM) in the presence or absence of collagen peptides for 24 h. Finally, cell viability was determined using the CCK-8 assay.

### 3.7. Anti-Inflammatory Activity Determination

C518 cells were seeded in 96-well plates (2 × 10^4^ cells/mL, 100 µL) and incubated for 24 h. They were pretreated with various collagen peptide samples (2.0 mg/mL) and cultured for 1 h. The control was pretreated with DMEM under the same conditions. Thereafter C518 cells were exposed to LPS (10 μg/mL) in the presence or absence of collagen peptides for 24 h. Finally, the abundance of IL-1β, PGE2, and TNF-α in cell-free supernatants was determined using ELISA kits according to the manufacturer’s instructions.

### 3.8. Spectrum-Effect Relationships

The spectrum-effect relationships between HPLC fingerprints and antioxidant and anti-inflammatory effects were established by GRA. Grey system theory is an interdisciplinary scientific area that was first introduced in the early 1980s [35], and it has been applied in decision making in an extremely wide range of multiple-attribute group decision-making problems [36].

#### 3.8.1. Data Preprocessing

In GRA, initial data preprocessing is performed to transform the original data sequences, with different measurement units, into comparable sequences. In this study, the mean value normalization preprocessing method was applied in the data series treatment to obtain a dimensionless matrix, because this method can easily accommodate a wide range of units among the factors used [37].

#### 3.8.2. Definition of Reference Sequences and Comparison Sequences

The antioxidant and anti-inflammatory activities of the collagen peptides were utilized as reference sequences, and the characteristic peak areas obtained by HPLC fingerprints were utilized as comparison sequences. The original reference sequences and comparison sequences are represented by {*X_o_*(*k*)} and {*X_i_*(*k*)}, *i* = 1, 2, …, *m*; *k* = 1, 2, …, *n* respectively, where *m* is the number of experiments and *n* is the total number of observations of data.

#### 3.8.3. Grey Relational Grade Calculation

The grey relational coefficient was calculated from the deviation sequence using the following relation:

(3) $$\gamma \left\{X_{o}\left(k\right),X_{i}\left(k\right)\right\}=\frac{\Delta \min+\xi \Delta \max}{\Delta_{oi}\min(k)+\xi \Delta \max}\;0<\gamma \{X_{o}\left(k\right),X_{i}\left(k\right)\}\ll 1,$$

where Δ*_oi_*(*k*) is the deviation sequence of the reference sequence {*X_o_*(*k*)} and comparability sequence {*X_i_*(*k*)}, and ξ is the resolution coefficient, usually ξ ∈ (0,1). The resolution coefficient is typically chosen to be 0.5. A grey relational grade is the weighted average of the grey relational coefficient and is defined as follows:

(4) $$\gamma_{oi}=\frac{1}{n}\gamma_{oi}\left\{X_{o}\left(k\right),X_{i}\left(k\right)\right\},$$

#### 3.8.4. Grey Relational Coefficient Calculation and Ranking

The grey relational grade between the reference sequence and comparison sequences was calculated. The grey relational grade was proportional to the similarity of the developing trends (i.e., the greater the grade, the more similarity) [38].

### 3.9. Purification and Identification of Collagen Peptides 

#### 3.9.1. Purification of Collagen Peptides by RP-HPLC

The collagen peptide was dissolved in distilled water and loaded onto a semipreparative C18 RP-HPLC column (10.0 mm × 250 mm, 5 μm, Agilent Technologies, Santa Clara, CA, USA). The HPLC 1200 system (Agilent Technologies, USA) was equipped with a quaternary pump solvent delivery system and a diode-array detector (DAD). The sample injection volume and concentration were 250 μL and 10 mg/mL, respectively. The column was eluted by a linear gradient of acetonitrile (2–60%) containing 0.1% TFA at a flow rate of 1.0 mL/min. The UV absorbance of the eluent was monitored at 214 nm. This step was repeated several times, and the different elution fractions were pooled, concentrated, and lyophilized for sequence identification.

#### 3.9.2. Identification of Collagen Peptides by UPLC-MS/MS Analysis

A Waters Acquity UPLC system (Waters, Milford, MA, USA) coupled with a Thermo Q Exactive mass spectrometer (Thermo Fisher Scientific, Bremen, Germany) was used for peptide separation and identification. The UPLC-MS/MS operating conditions were as follows: The column was an Eksigent C18 trap column (75 µm × 250 mm, 3 μm). A binary gradient elution system, comprising (A) 0.1% TFA/acetonitrile and (B) 0.1% TFA/water, was applied as follows: Initial, 90% B; 60 min, 40% B. The detection wavelengths were 214 and 280 nm, the flow rate was 3.0 μL/min, the freeze-dried sample was dissolved in 0.1% aqueous formic acid, and the injection volume was 1.0 μL. The mass spectrometer was fitted with an electrospray ionization (ESI) source used in the positive ion mode.

### 3.10. Statistical Analysis

All assays were carried out using at least three independent sets of experiments, and all results were expressed as average values with their corresponding standard deviations. One-way analysis of variance by Tukey’s test was performed using SPSS 22.0 (IBM Co., Armonk, NY, USA).

## 4. Conclusions

In this work, HPLC fingerprints and a series of in vitro antioxidant and anti-inflammatory assays were combined to investigate the spectrum-effect relationship of collagen peptides. The results showed that peaks 2, 3, and 8 were the main components contributing to the antioxidant activity of the collagen peptides, whereas peaks 11 and 14 were the main components contributing to the anti-inflammatory activity of the collagen peptides. Subsequently, the amino acid sequences of peaks 8 and 14 were identified as GPRGPPGPVGP and VAIQAVLSLYASGR by UPLC-MS/MS. These identified peptides may have potential as drugs or functional foods for the treatment or prevention of OA. This report establishes a new platform for identifying the functional components of collagen peptides by the spectrum-effect relationship, which may lead to the development of new directions in the utilization of bioactive peptides in the future. However, further studies are required to investigate the cellular and molecular mechanism by which peptide pretreatment exerts its effects in the animal OA models.

## Figures and Tables

**Table 1:** The relative retention time (RRT) and relative peak area (RPA) of the common peaks of the collagen peptides. C.V.%: coefficient of variance.

Peak No.	RRT	S1	S2	S3	S4	S5	S6	S7	S8	S9	S10	S11	S12	S13	C.V.%
1	0.538	0.333	0.376	0.241	0.329	0.307	0.364	0.372	0.355	0.400	0.371	0.348	0.337	0.499	18.54
2	0.620	4.766	4.808	5.099	4.516	4.653	4.700	4.821	4.745	5.031	4.780	4.769	4.543	1.761	22.17
3	0.669	3.585	3.401	4.693	3.406	3.429	3.467	3.502	3.478	3.449	3.420	3.535	3.395	2.997	13.60
4	0.720	0.533	0.617	0.453	0.570	0.559	0.594	0.608	0.590	0.750	0.625	0.527	0.575	0.880	20.92
5	0.768	0.182	0.201	0.098	0.183	0.156	0.178	0.193	0.191	0.226	0.209	0.180	0.176	0.000	37.45
6	0.789	2.972	2.862	2.683	2.828	2.825	2.857	2.893	2.898	3.043	2.964	2.950	2.834	0.081	31.60
7	0.875	0.751	0.547	0.791	0.664	0.845	0.675	0.696	0.598	0.315	0.414	0.703	0.655	0.000	41.04
8(s)	1.000	1.000	1.000	1.000	1.000	1.000	1.000	1.000	1.000	1.000	1.000	1.000	1.000	1.000	9.95
9	1.266	0.198	0.179	0.149	0.177	0.177	0.105	0.141	0.207	0.192	0.185	0.182	0.163	0.256	22.45
10	1.308	0.655	0.440	0.343	0.408	0.473	0.403	0.402	0.426	0.305	0.360	0.652	0.394	0.170	34.07
11	1.629	0.451	0.372	0.229	0.438	0.304	0.364	0.348	0.350	0.246	0.295	0.445	0.350	0.183	26.69
12	1.957	1.459	0.923	0.618	0.928	0.866	0.862	0.932	0.893	0.651	0.805	1.396	0.934	0.261	36.93
13	2.044	0.045	0.253	0.359	0.304	0.218	0.304	0.357	0.323	0.233	0.371	0.383	0.358	0.386	27.75
14	2.216	1.086	0.693	0.665	0.709	0.539	0.602	0.719	0.703	0.511	0.741	0.751	0.694	0.608	23.95
15	2.316	0.415	0.269	0.258	0.270	0.221	0.233	0.280	0.275	0.219	0.282	0.277	0.264	0.288	22.00

**Table 2:** Similarities among collagen peptides from 13 origins.

	S1	S2	S3	S4	S5	S6	S7	S8	S9	S10	S11	S12	S13	R
S1	1.000													
S2	0.704	1.000												
S3	0.747	0.950	1.000											
S4	0.733	0.964	0.984	1.000										
S5	0.695	0.926	0.959	0.959	1.000									
S6	0.696	0.889	0.932	0.921	0.969	1.000								
S7	0.691	0.877	0.920	0.909	0.963	0.998	1.000							
S8	0.696	0.888	0.928	0.918	0.967	0.972	0.967	1.000						
S9	0.687	0.884	0.924	0.912	0.964	0.987	0.985	0.972	1.000					
S10	0.680	0.870	0.910	0.899	0.957	0.963	0.961	0.994	0.974	1.000				
S11	0.696	0.885	0.916	0.910	0.918	0.927	0.916	0.950	0.919	0.940	1.000			
S12	0.689	0.868	0.911	0.903	0.909	0.917	0.907	0.939	0.913	0.932	0.993	1.000		
S13	0.626	0.612	0.693	0.637	0.631	0.642	0.635	0.639	0.637	0.633	0.702	0.714	1.000	
R	0.777	0.936	0.972	0.963	0.977	0.976	0.969	0.977	0.972	0.967	0.961	0.953	0.711	1.000

**Table 3:** The RRT and RPA of the common peaks of the amino acids.

Peak No.	RRT	S1	S2	S3	S4	S5	S6	S7	S8	S9	S10	S11	S12	S13	C.V.%
1	0.361	0.944	0.903	0.885	0.953	0.974	0.843	0.865	0.854	0.804	0.896	0.857	0.915	0.931	8.30
2	0.413	1.701	1.646	1.712	1.732	1.800	1.645	1.648	1.622	1.555	1.627	1.612	1.717	1.775	8.63
3	0.603	1.804	1.801	1.905	1.893	1.918	2.020	2.015	1.966	2.013	2.008	2.089	2.101	1.939	12.48
4	0.801	0.639	0.618	0.607	0.632	0.796	0.709	0.721	0.709	0.663	0.680	0.680	0.689	0.704	12.10
5	0.861	6.032	5.889	6.115	6.099	6.320	6.367	6.329	6.125	6.366	6.308	6.569	6.575	6.287	10.86
6	0.894	0.134	0.133	0.121	0.135	0.157	0.134	0.139	0.137	0.131	0.133	0.145	0.135	0.143	9.29
7(s)	1.000	1.000	1.000	1.000	1.000	1.000	1.000	1.000	1.000	1.000	1.000	1.000	1.000	1.000	10.39
8	1.075	0.631	0.611	0.599	0.626	0.705	0.650	0.659	0.641	0.627	0.632	0.666	0.656	0.661	9.96
9	1.098	0.065	0.061	0.068	0.067	0.063	0.058	0.052	0.050	0.052	0.057	0.064	0.070	0.081	10.37
10	1.125	2.148	2.076	2.126	2.137	2.204	2.096	2.114	2.047	2.082	2.083	2.120	2.136	2.143	9.40
11	1.156	2.398	2.372	2.440	2.441	2.539	2.529	2.524	2.461	2.566	2.551	2.683	2.661	2.549	10.60
12	1.263	1.638	1.608	1.948	1.833	2.146	1.391	1.322	1.339	1.443	1.556	1.545	1.926	1.974	10.40
13	1.649	0.212	0.197	0.173	0.191	0.205	0.182	0.187	0.188	0.181	0.187	0.216	0.198	0.152	12.71
14	1.741	0.732	0.665	0.627	0.670	0.736	0.636	0.641	0.609	0.590	0.601	0.662	0.639	0.624	9.65
15	1.809	0.328	0.328	0.334	0.361	0.379	0.335	0.348	0.343	0.354	0.335	0.353	0.355	0.328	10.43
16	1.938	0.479	0.481	0.398	0.455	0.498	0.448	0.453	0.429	0.425	0.021	0.484	0.462	0.438	30.24
17	1.957	0.943	0.920	0.833	0.922	0.982	0.914	0.936	0.905	0.897	0.426	0.969	0.944	0.943	18.25
18	2.027	0.366	0.345	0.349	0.357	0.394	0.448	0.445	0.442	0.252	0.008	0.289	0.255	0.318	40.55
19	2.045	0.111	0.110	0.125	0.128	0.135	0.147	0.126	0.123	0.079	0.284	0.101	0.104	0.144	39.35
20	2.061	0.476	0.464	0.443	0.474	0.528	0.480	0.485	0.472	0.470	0.473	0.515	0.513	0.447	10.51
21	2.166	1.026	0.985	0.949	0.985	1.090	0.942	0.966	0.951	0.955	0.951	1.003	0.971	1.059	7.48

**Table 4:** Similarities among amino acids from 13 origins.

	S1	S2	S3	S4	S5	S6	S7	S8	S9	S10	S11	S12	S13	R
S1	1.000													
S2	1.000	1.000												
S3	0.999	0.999	1.000											
S4	0.999	1.000	1.000	1.000										
S5	0.998	0.998	0.999	0.999	1.000									
S6	0.998	0.998	0.997	0.997	0.995	1.000								
S7	0.998	0.998	0.996	0.997	0.995	1.000	1.000							
S8	0.998	0.998	0.996	0.997	0.995	1.000	1.000	1.000						
S9	0.998	0.998	0.997	0.997	0.995	0.999	0.999	0.999	1.000					
S10	0.999	0.999	0.998	0.999	0.997	0.999	0.999	0.999	1.000	1.000				
S11	0.998	0.998	0.997	0.998	0.996	0.999	0.999	0.999	1.000	1.000	1.000			
S12	0.998	0.999	0.999	0.999	0.998	0.998	0.997	0.998	0.999	0.999	0.999	1.000		
S13	0.999	0.999	1.000	1.000	0.999	0.997	0.996	0.997	0.997	0.998	0.998	0.999	1.000	
R	0.999	1.000	0.999	0.999	0.998	0.999	0.999	0.999	0.999	1.000	0.999	0.999	0.999	1.000

**Table 5:** Molecular weight distribution of collagen peptides.

Samples	<500 (Da)	500–1000 (Da)	1000–3000 (Da)	>3000 (Da)
S1	12.48	45.32	24.60	17.60
S2	8.89	39.18	30.77	21.16
S3	9.68	30.68	26.23	33.41
S4	12.89	32.34	25.76	29.01
S5	13.09	32.99	20.99	32.93
S6	12.37	34.21	22.21	31.21
S7	7.56	34.10	32.03	26.31
S8	7.03	33.55	30.48	28.94
S9	8.90	43.10	31.82	16.18
S10	9.51	44.95	24.41	21.13
S11	12.51	43.36	28.25	15.88
S12	10.24	42.43	31.54	15.79
S13	6.07	27.28	29.76	36.89

**Table 6:** The antioxidant activity of collagen peptides in the 2,2′-Azinobis (3-ethylbenzothiazoline-6-sulphonic acid) diammonium salt (ABTS) assay, the 2,2-diphenyl-1-picrylhydrazyl (DPPH) assay, and the ferric-reducing antioxidant power (FRAP) assay.

Samples	ABTS	DPPH	FRAP
S1	48.14 ± 0.15	44.38 ± 1.28	37.02 ± 0.57
S2	43.21 ± 0.19	45.94 ± 2.50	35.19 ± 0.72
S3	36.78 ± 0.25	40.16 ± 1.17	42.65 ± 1.00
S4	42.65 ± 1.23	46.05 ± 2.18	33.06 ± 0.51
S5	43.30 ± 1.38	53.50 ± 4.36	31.56 ± 0.17
S6	41.93 ± 0.43	49.83 ± 2.10	37.65 ± 0.22
S7	46.07 ± 0.39	51.50 ± 1.12	34.77 ± 0.34
S8	44.72 ± 0.17	49.28 ± 2.93	33.83 ± 0.25
S9	38.79 ± 0.35	50.39 ± 2.06	45.58 ± 0.54
S10	42.50 ± 0.37	53.62 ± 1.21	49.10 ± 0.56
S11	47.84 ± 0.21	54.23 ± 2.39	38.08 ± 0.47
S12	43.28 ± 0.18	55.06 ± 2.49	34.00 ± 0.35
S13	34.91 ± 0.22	18.46 ± 1.64	18.15 ± 0.90

**Table 7:** Inhibitory effects of collagen peptides on lipopolysaccharide (LPS)-induced IL-1β, TNF-α, and PGE2 release in C518 cells.

Group	IL-1β (ng/L)	TNF-α (ng/L)	PGE2 (ng/L)
Control	69.66 ± 4.32	169.65 ± 6.85	90.35 ± 5.15
LPS-treated	86.62 ± 2.44 ^#^	211.33 ± 11.43 ^#^	115.10 ± 5.20 ^#^
S1	61.69 ± 1.37 **	152.62 ± 7.55 *	40.79 ± 9.56 **
S2	44.42 ± 0.81 **	128.97 ± 3.09 *	47.10 ± 2.81 **
S3	78.73 ± 1.28	132.91 ± 10.76 *	59.56 ± 0.02 **
S4	81.94 ± 0.28	172.71 ± 14.85 *	52.57 ± 5.22 **
S5	79.22 ± 1.84	168.35 ± 8.69 *	74.69 ± 2.90 **
S6	85.00 ± 2.99	158.69 ± 5.57 *	49.68 ± 2.81 **
S7	75.99 ± 2.51	199.97 ± 0.78 *	55.84 ± 4.92 **
S8	69.37 ± 4.56 *	193.53 ± 8.59 *	40.79 ± 9.97 **
S9	72.15 ± 5.55 *	185.42 ± 7.68 *	52.57 ± 7.47 **
S10	42.46 ± 4.70 **	176.09 ± 1.32 *	55.84 ± 4.92 **
S11	54.56 ± 2.10 **	193.05 ± 5.25 *	49.68 ± 6.29 **
S12	59.48 ± 3.28 **	184.95 ± 6.76 *	52.57 ± 9.80 **
S13	74.5 ± 3.15 *	182.56 ± 7.17 *	68.82 ± 2.89 **

Data are expressed as means ± SD (*n* = 3); ** *p* < 0.01 and * *p* < 0.05 versus the LPS-treated group; ^#^ *p* < 0.05 versus the control group.

**Table 8:** The correlation coefficient between the common characteristic peaks and the efficacy of collagen peptides.

Peak No.	r						
	ABTS	DPPH	FRAP	Cell Viability	IL-1β	TNF-α	PGE2
1	0.7732	0.7338	0.7722	0.7315	0.6441	0.6492	0.7622
2	0.7788	0.8110	0.8102	0.7334	0.6435	0.6733	0.8079
3	0.7795	0.7744	0.8081	0.7065	0.6531	0.7025	0.7983
4	0.7497	0.7451	0.7584	0.7294	0.6303	0.6537	0.7368
5	0.7503	0.7467	0.6933	0.7168	0.6450	0.6313	0.7748
6	0.7795	0.7853	0.7456	0.7166	0.6349	0.6501	0.8282
7	0.6673	0.7068	0.6722	0.6819	0.6279	0.6617	0.7029
8	0.8040	0.7982	0.7783	0.7085	0.6507	0.6852	0.8159
9	0.7285	0.7181	0.6870	0.7345	0.6569	0.6555	0.7486
10	0.7008	0.7639	0.6960	0.7534	0.6802	0.6574	0.7271
11	0.7225	0.7461	0.6985	0.7517	0.6943	0.6827	0.7707
12	0.7161	0.7728	0.6950	0.7331	0.6811	0.6581	0.7486
13	0.7544	0.7842	0.7083	0.6555	0.6238	0.6039	0.7446
14	0.8080	0.7530	0.7561	0.7428	0.6729	0.6853	0.8254
15	0.7947	0.7428	0.7397	0.7492	0.6587	0.6789	0.7932

**Table 9:** The processing conditions of collagen peptides. E/S: enzyme to substrate ratio.

Samples	E/S (*w*/*w*)	pH	Temperature (°C)	Time (h)
S1	0.75%	6.0	50	6
S2	0.50%	6.0	50	6
S3	0.25%	6.0	50	6
S4	0.50%	6.0	55	6
S5	0.50%	6.5	55	6
S6	0.50%	7.0	55	6
S7	0.50%	7.0	50	4
S8	0.50%	7.0	55	4
S9	0.50%	7.0	60	4
S10	0.50%	6.5	50	6
S11	0.50%	6.5	50	5
S12	0.50%	6.5	50	4
S13	0.25%	6.5	50	6

## Supplementary Materials

The following are available online, Figures S1–S13: The HPLC fingerprints of collagen peptide samples S1–S13.
