# Supplementary material for: Spectrum-Effect Relationships between High-Performance Liquid Chromatography (HPLC) Fingerprints and the Antioxidant and Anti-Inflammatory Activities of Collagen Peptides
Source: Molecules. 2018 Dec 10;23(12):3257. doi: 10.3390/molecules23123257 (PMC6320860; doi:10.3390/molecules23123257)
Supplement: Supplementary file 1 [file molecules-23-03257-s001.pdf]

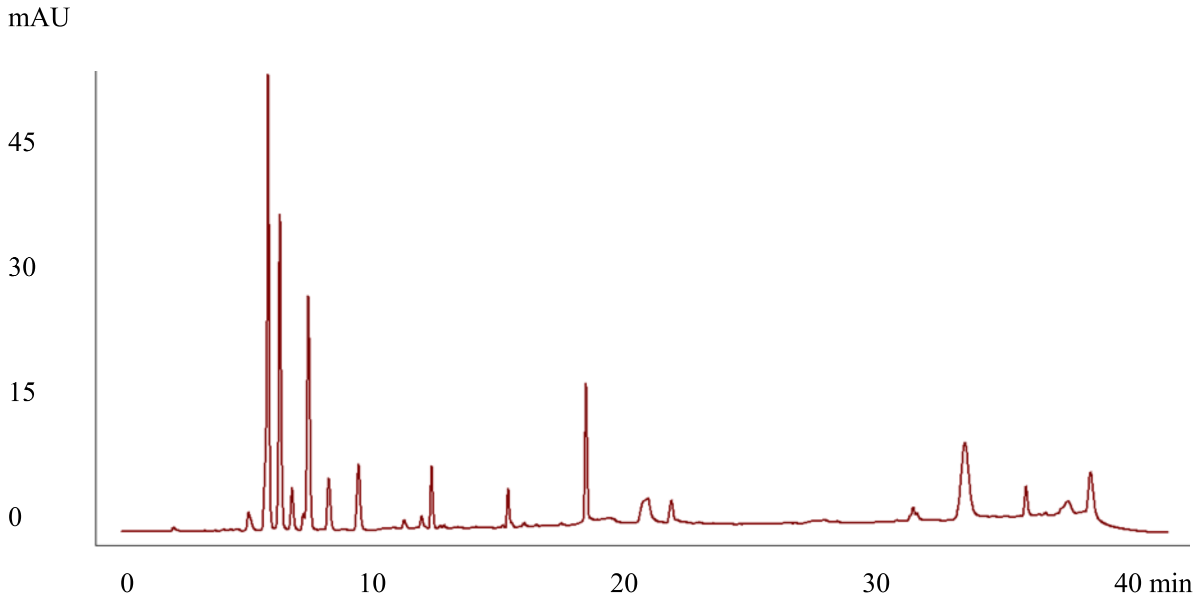

Figure S1. The HPLC fingerprints of collagen peptide sample S1

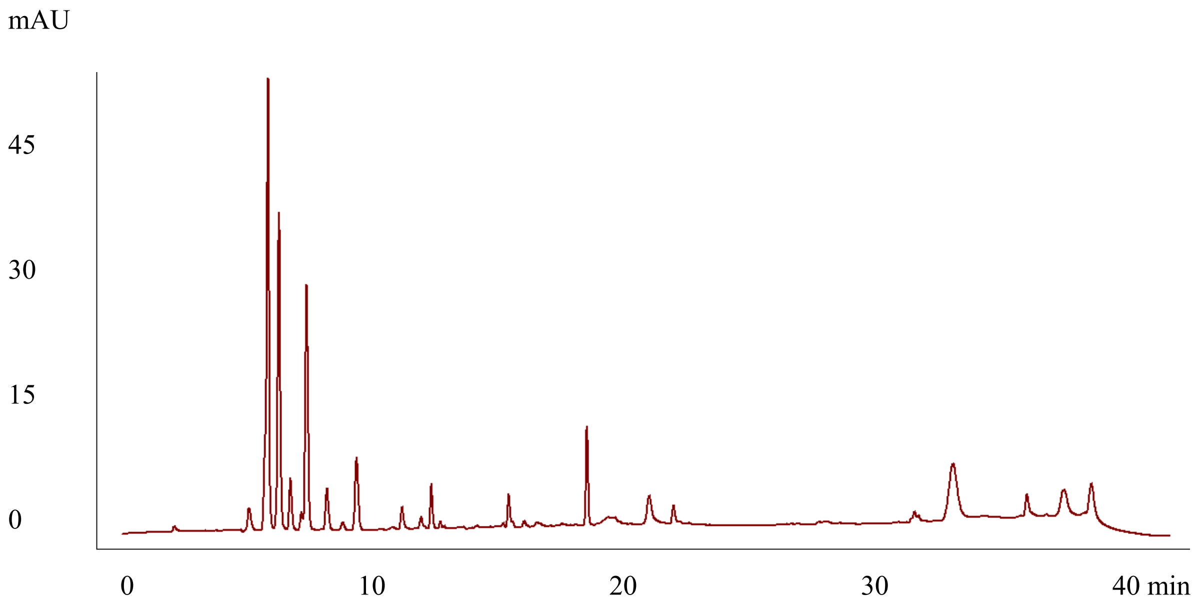

Figure S2. The HPLC fingerprints of collagen peptide sample S2

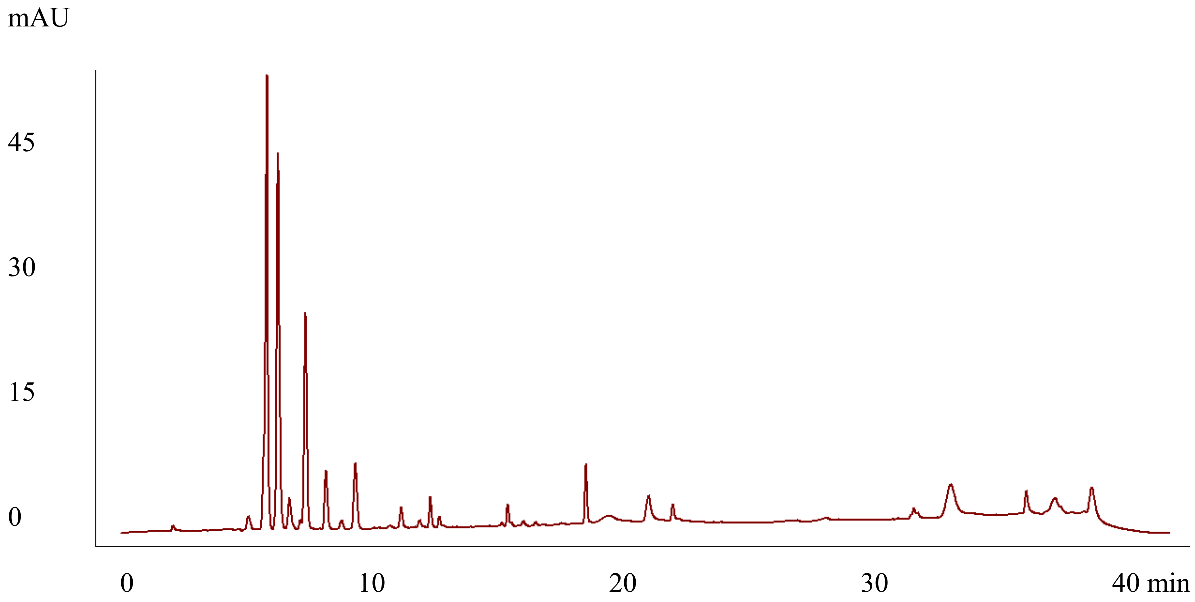

Figure S3. The HPLC fingerprints of collagen peptide sample S3

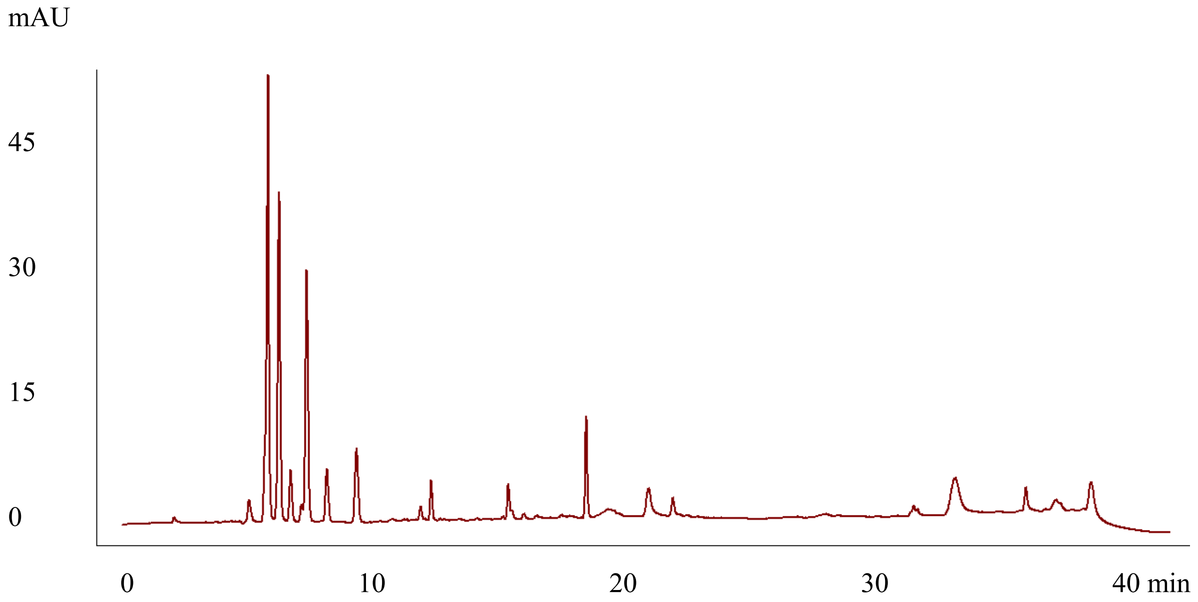

Figure S4. The HPLC fingerprints of collagen peptide sample S4

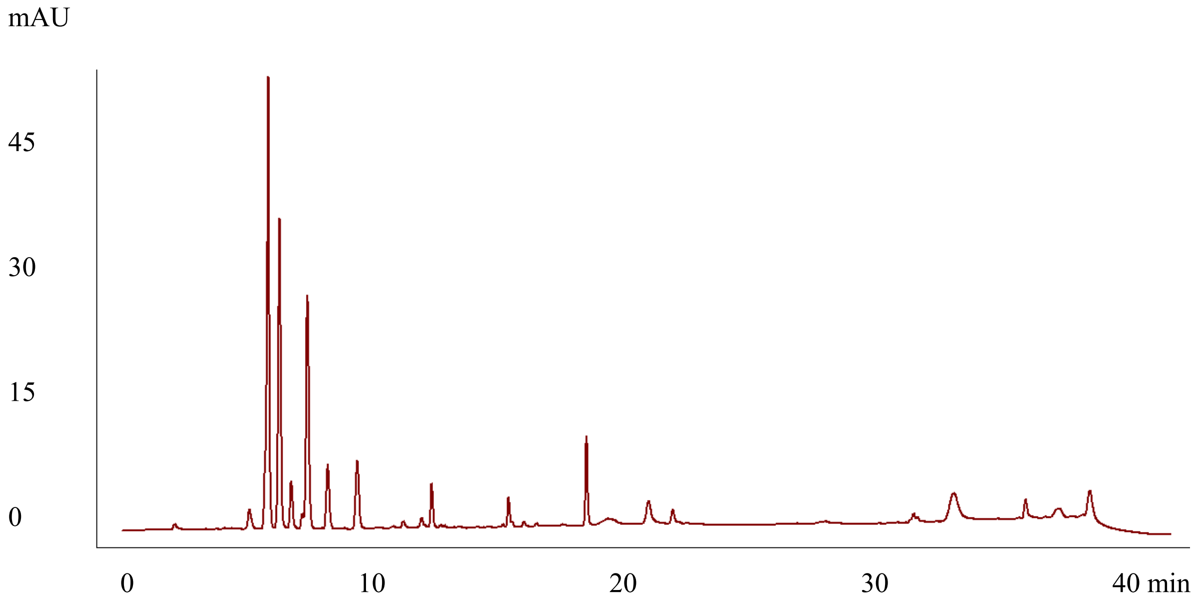

Figure S5. The HPLC fingerprints of collagen peptide sample S5

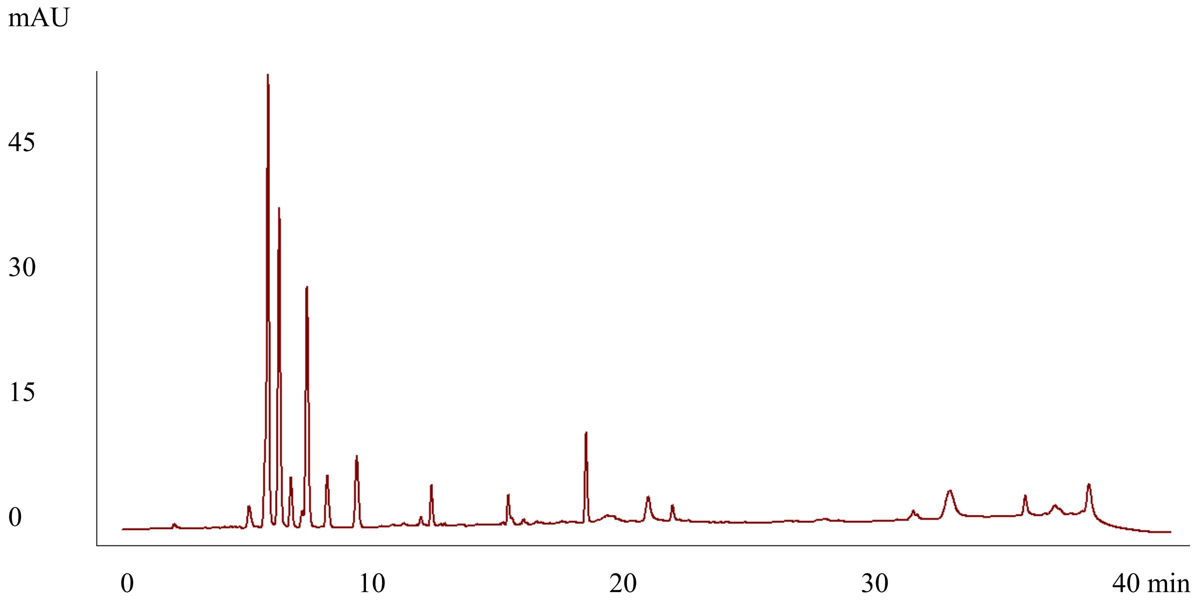

Figure S6. The HPLC fingerprints of collagen peptide sample S6

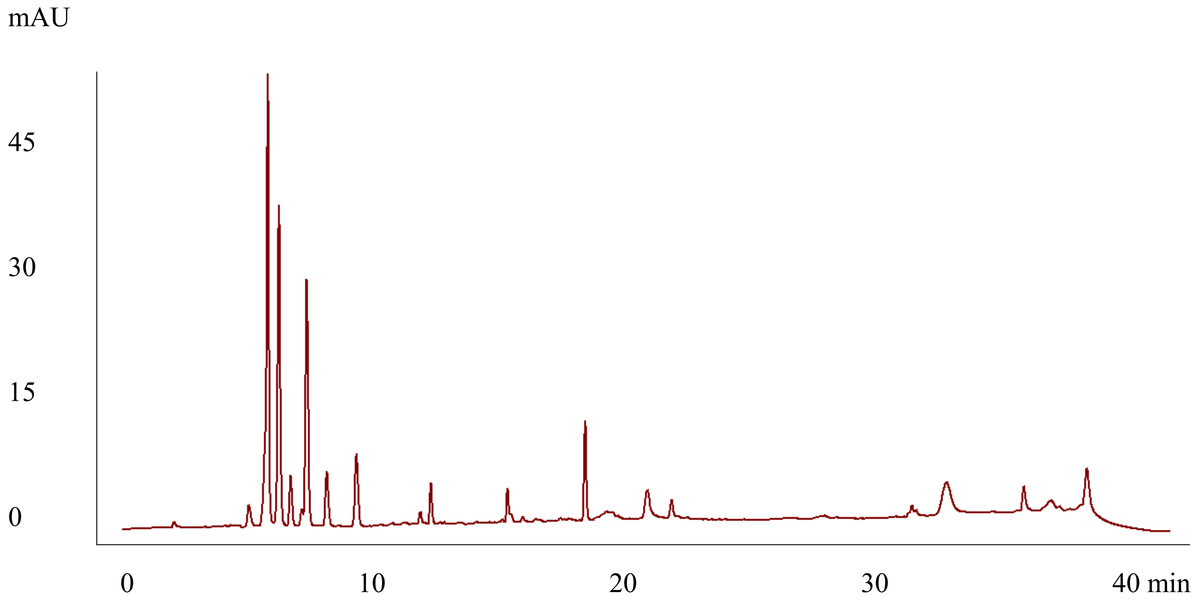

Figure S7. The HPLC fingerprints of collagen peptide sample S7

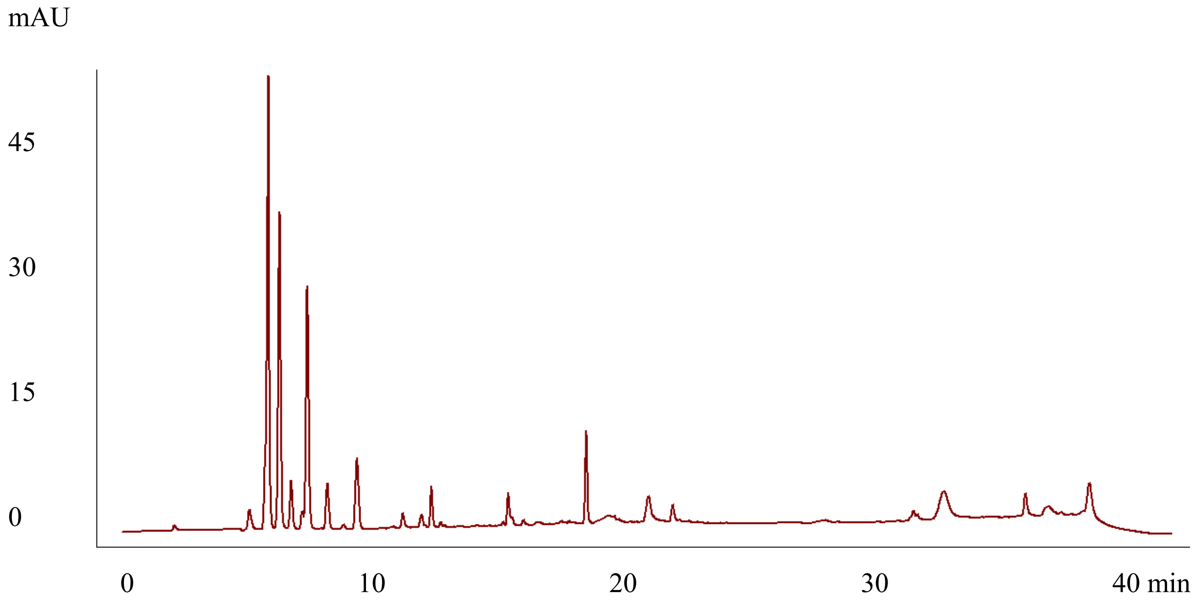

Figure S8. The HPLC fingerprints of collagen peptide sample S8

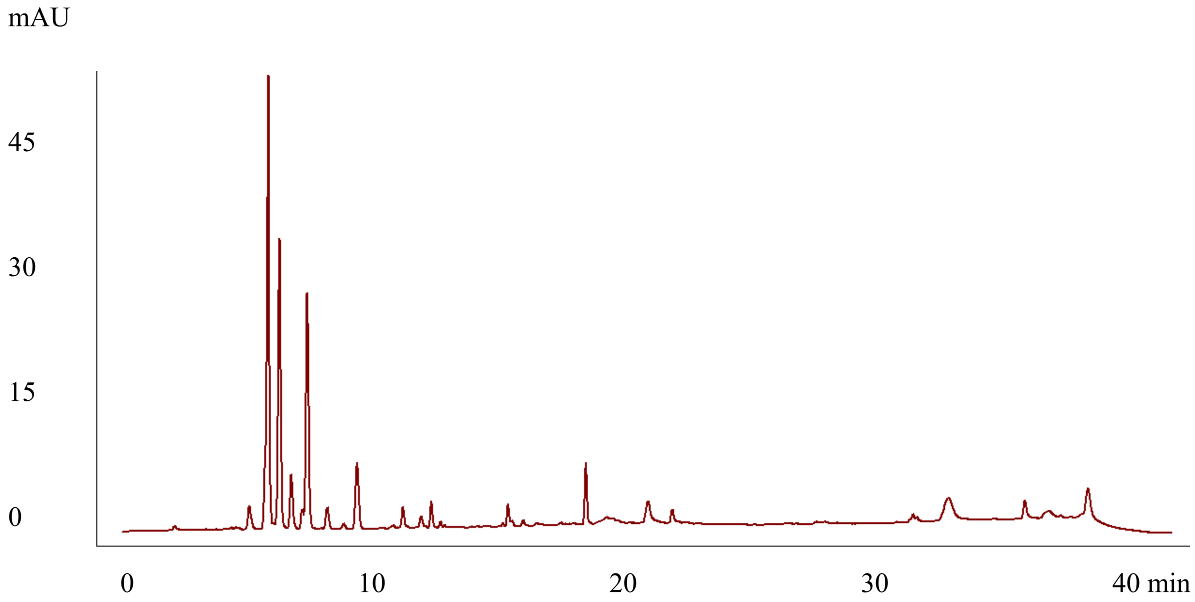

Figure S9. The HPLC fingerprints of collagen peptide sample S9

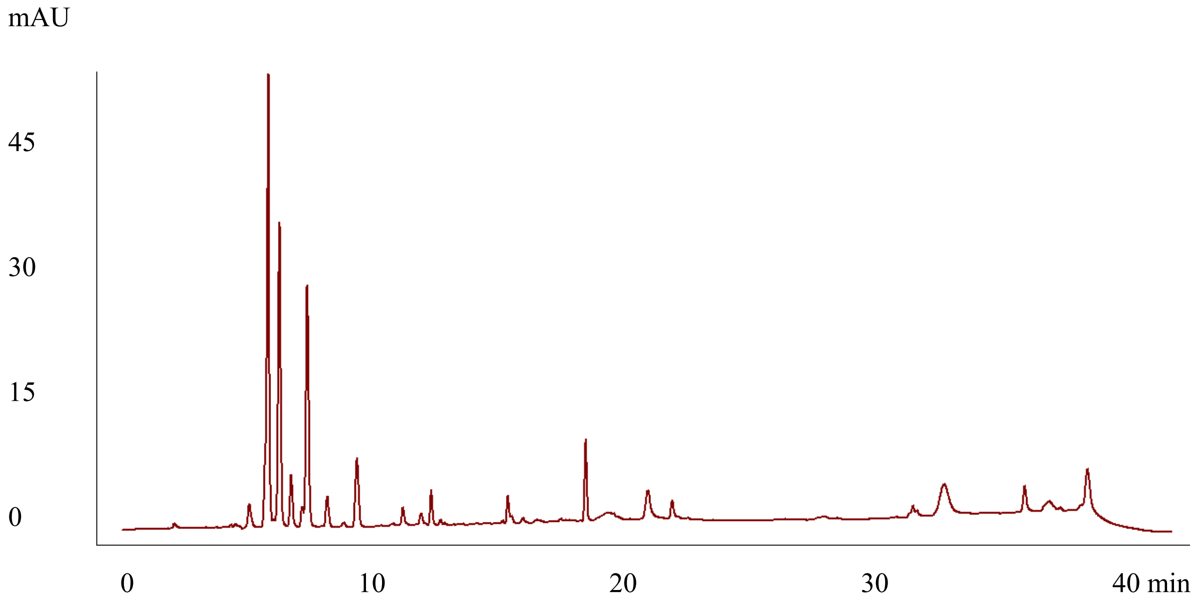

Figure S10. The HPLC fingerprints of collagen peptide sample S10

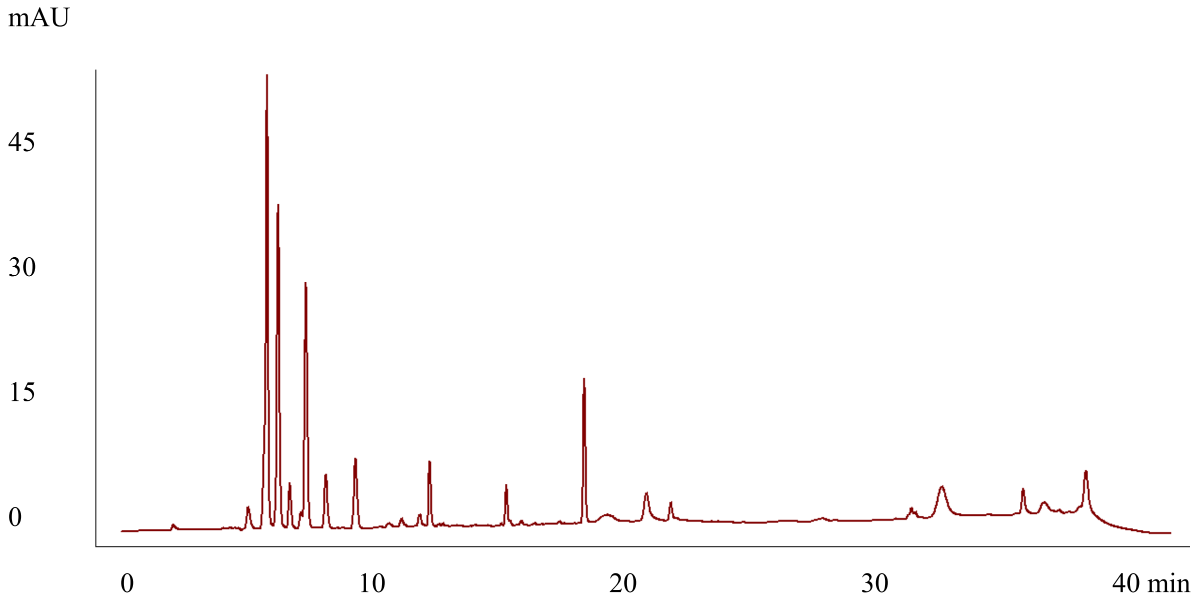

Figure S11. The HPLC fingerprints of collagen peptide sample S11

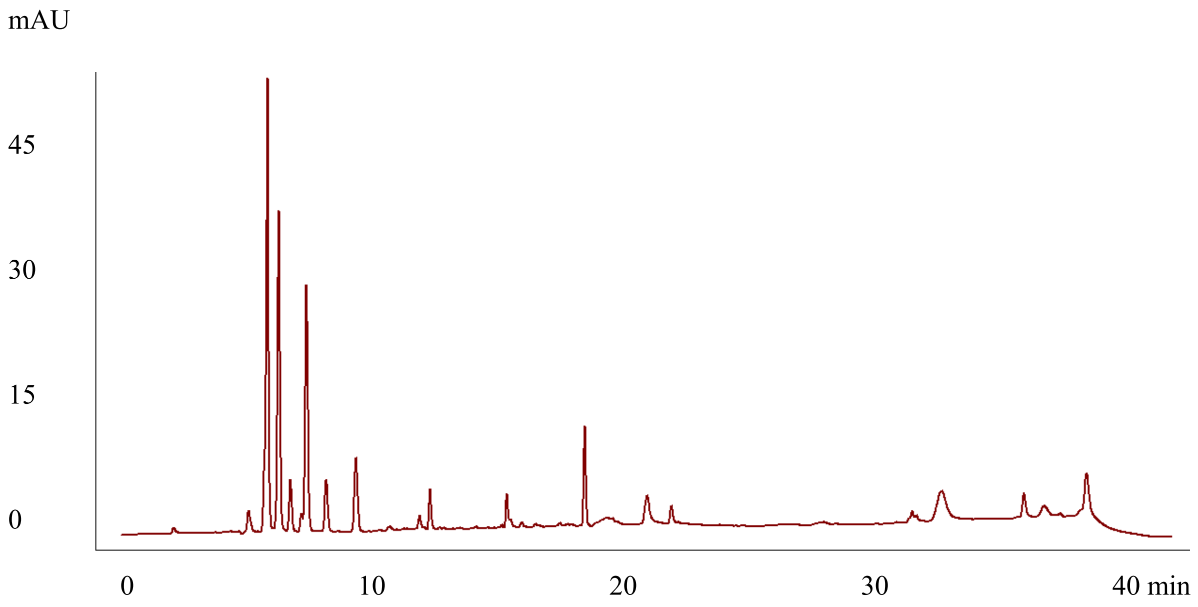

Figure S12. The HPLC fingerprints of collagen peptide sample S12

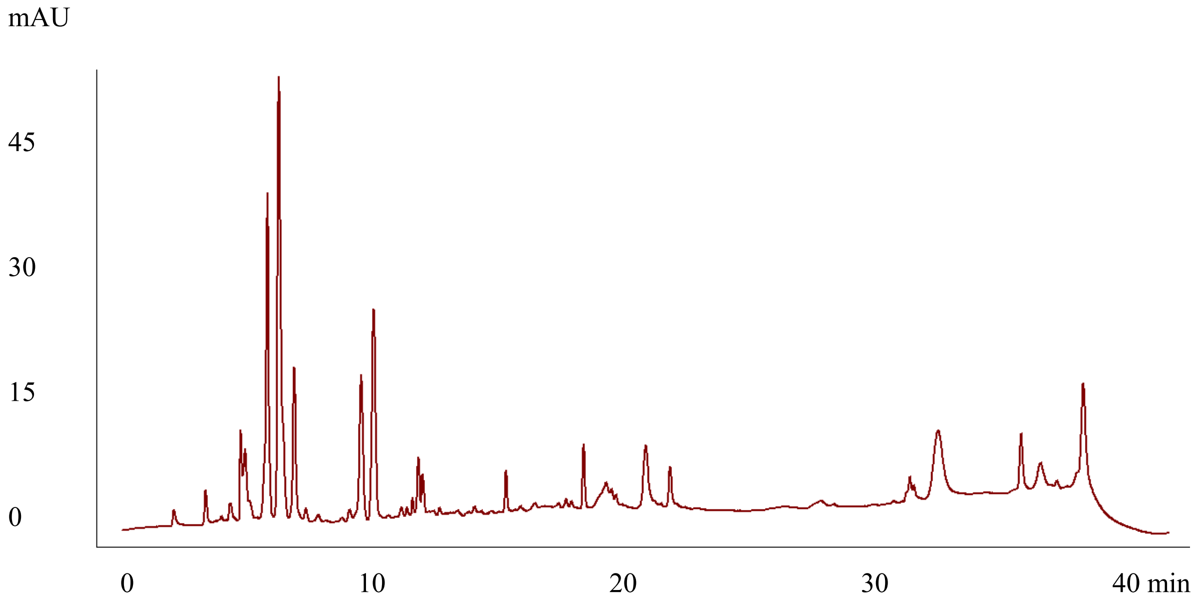

Figure S13. The HPLC fingerprints of collagen peptide sample S13
